# Hydrothermal Treatment with Different Solvents for Composite Recycling and Valorization Under Subcritical and Supercritical Conditions

**DOI:** 10.3390/polym18010089

**Published:** 2025-12-28

**Authors:** José M. Vázquez-Fernández, Belén García-Jarana, Milagrosa Ramírez-del Solar, Lucio Cardozo-Filho, Juan R. Portela-Miguélez, José M. Abelleira-Pereira

**Affiliations:** 1 Department of Chemical Engineering and Food Technology, Faculty of Sciences, University of Cádiz, International Excellence Agrifood Campus (CeiA3), Puerto Real, 11510 Cádiz, Spain; josemanuel.vazquez@uca.es (J.M.V.-F.); belen.garcia@uca.es (B.G.-J.); juanramon.portela@uca.es (J.R.P.-M.); 2 Department of Condensed Matter Physics, Faculty of Sciences, University of Cádiz, Campus Universitario Puerto Real, Puerto Real, 11510 Cádiz, Spain; milagrosa.ramirez@uca.es; 3 Programa de Pós-Graduação em Engenharia Química, Universidade Estadual de Maringá (UEM), Avenida Colombo, 5790–Zona 7, Maringá 87020-900, PR, Brazil; lcfilho@uem.br; 4 Department of Chemical Engineering and Food Technology, Higher Technical School of Engineering of Algeciras (ETSIA), University of Cádiz, Algeciras, 11202 Cádiz, Spain

**Keywords:** hydrothermal treatment, subcritical water, supercritical water, waste valorization, aeronautical wastes, composites valorization, carbon fibers reclamation, catalyst, H_2_ production

## Abstract

Worldwide, carbon fiber (CF) demand has been rising over the last decade, which contrasts with the fact that up to 30–50% of composite materials in aircraft production are scrapped. This situation highlights the increasing need for recycling methods to reduce fabrication costs and global warming potential. Emerging technologies focus on recovering long CFs, as they represent the most valuable form but are also the most difficult to reclaim using conventional recycling methods. Hydrothermal treatments offer a promising alternative to valorize this waste by decomposing the polymer matrix under subcritical and supercritical conditions without significantly damaging the fibers. Water, isopropanol, and mixtures of water/isopropanol or water/acetone were tested as solvents, with and without the addition of zinc chloride (ZnCl_2_) as a homogeneous catalyst. The influence of temperature, pressure, and solvent composition on resin degradation was evaluated. In this work, degradation rates of up to 92% were achieved at 415 °C, 233 bar, 120 min, 5 wt.% IPA, and ZnCl_2_ 0.1 M. It should be noted that ZnCl_2_ caused reactor corrosion. Furthermore, the recovered fibers retained their morphology, including the sizing layer, and showed mechanical properties similar to the original material, while a small H_2_-rich gaseous fraction was generated as a byproduct of the hydrothermal degradation. Using water–isopropanol solutions resulted in the reactor being significantly cleaner than when using water alone, which can be advantageous for future scale-up and for reducing maintenance requirements. These results confirm the potential of hydrothermal processing as an efficient and selective method for the recycling and valorization of carbon-fiber-reinforced composites from the aeronautical industry.

## 1. Introduction

According to the literature, global demand for carbon fibers (CFs) across multiple application sectors has increased markedly over the past decade. Moreover, 30–50% of composite materials used in aircraft production are scrapped as a consequence of current manufacturing practices. European Union restrictions on the landfilling of organic waste, together with the associated disposal costs for scrapped composite materials, are driving the increased adoption of recycling methods [1,2]. Reclaiming CFs and reusing them in new composite materials can significantly reduce both manufacturing costs and the global warming potential. Virgin CF products typically sell for EUR 20–40/kg, whereas recycled fibers are generally priced at EUR 10–20/kg [3,4]. Emerging technologies prioritize the recovery of long CFs, as this is the highest-value form [5] and is more difficult to obtain using conventional recycling approaches. Hydrothermal hydrolysis/solvolysis can successfully valorize composite off-cuts by dissolving the resin and recovering the carbon fibers. After fiber recovery, the aqueous effluent containing dissolved resin may be further exploited for energy recovery via hydrothermal liquefaction or supercritical water gasification. This work studies the hydrothermal treatment for the recycling and valorization of aeronautical composite off-cuts. To contextualize the origin and nature of the materials targeted for recovery, key fundamentals of carbon-fiber production and composite manufacturing are briefly summarized.

### 1.1. Manufacturing of CFs and Composites

Using polyacrylonitrile (PAN) thermoplastic fibers as precursors, high-quality carbon fibers can be manufactured. First, the PAN fibers are thermally oxidized in air at 200–400 °C and then carbonized at around 1000 °C; if required for the intended application, they are graphitized at higher temperatures (up to ≈3000 °C) [6]. Finally, after surface treatment and epoxy sizing, the resulting high-quality carbon fibers are ready for use. The carbon fibers are then pre-impregnated with an epoxy matrix to produce pre-preg layers, which are stacked and placed into vacuum bags. Eventually, this laminate lay-up goes through a curing process in an autoclave at, for instance, 120–180 °C, yielding the final composite material.

The European carbon fiber market is expected to grow significantly over the coming years. In fact, globally, it is expected to grow from around EUR 3.19 billion in 2024 to EUR 5.15–8.28 billion by 2031 [7,8]. The main applications include aviation, aerospace and defense, automotive, wind turbines, sports, construction, and others (e.g., medical devices). Virgin carbon fibers for general-purpose applications typically range from EUR 20 to EUR 40 per kilogram, whereas aerospace-grade fibers can cost between 160 and EUR 240 per kilogram [9]. According to another reference in the literature, recycled carbon fibers are estimated to cost between EUR 16 and EUR 23 per kilogram [10], although these are generally shorter, chopped or milled fibers with lower mechanical performance. In contrast, fibers recovered through hydrothermal treatment can retain the original length and exhibit mechanical properties comparable to those of virgin fibers and are, therefore, expected to reach a higher market value. Taken together, increasingly stringent legislation, rising costs associated with the landfilling of organic materials, the aforementioned global growth in CF demand, and the high energy intensity of CF production at 183–286 MJ/kg [11] are driving interest in developing more efficient and sustainable technologies to reclaim carbon fibers from difficult-to-recycle composite materials.

### 1.2. Carbon Fiber Composites Recycling

Most of the processes currently proposed for the recycling of carbon fiber-reinforced materials can be classified into three main groups: mechanical, thermal and chemical. The mechanical treatments (crushing, grinding, milling, etc.) are simple, but sometimes require the consumption of large amounts of energy and produce short fibers with reduced mechanical properties; therefore, they are generally unsuitable for remanufacturing and have a low market value [12]. Thermal treatments generally involve pyrolysis or fluidized-bed gasification (with air or steam), using high temperatures to break down the resin matrix and recover the carbon fibers. They have the advantage of not requiring chemical reagents or solvents. Pyrolysis is currently the most developed and widely implemented recycling process on an industrial scale, but the recovered fibers are generally degraded, with damaged surface morphology [13]. Chemical processes (including thermochemical approaches) involve the use of reagents (strong acids or bases, oxidants, catalysts, etc.) or solvents (water, alcohols, ketones, etc.) to decompose or dissolve the resin matrix and recover the carbon fibers. Among these methods, solvolysis (using solvents or heated solvents) has gained considerable attention as a possible economical technology to effectively reclaim and recover valuable high length carbon fibers. Due to the availability of solvents in combination with the use of different temperatures and catalysts, solvolysis offers many ways of recycling carbon fibers. A broad range of solvolysis processes is being intensively investigated to operate under milder conditions and with lower environmental impact, while preserving fiber mechanical properties and maximizing the performance of remanufactured composites.

**Figure 1:**
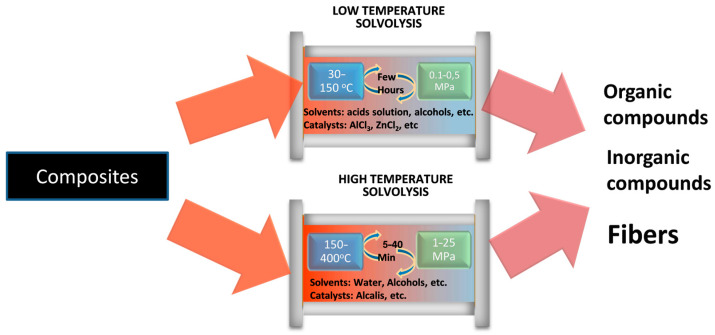
Types of solvolysis for carbon fiber reinforced polymer (CFRP) recycling.

Solvolysis uses reactive solvents, such as water, alcohols, ketones, ammonia, and glycol, to recycle composite materials by breaking down thermoset polymer matrix while recovering the fibers [14]. This chemical process can produce long and clean fibers (i.e., with no residue on the surface) with a minor or negligible reduction in their mechanical properties and can, therefore, yield high-quality recycled CFs. As shown in Figure 1, the following two main types of solvolysis can be found: low-temperature solvolysis, which typically operates at ambient pressure and the lowest feasible temperature to minimize the process energy demand; and high-temperature solvolysis, which increases recycling yields and reduces processing time by operating at elevated temperatures. The main results obtained by different researchers for both types of solvolysis are described below.

#### 1.2.1. Low-Temperature Solvolysis

Solvolysis with low-temperature and low-pressure processes presents some unique advantages, such as energy saving, simplified equipment, and limited or no damage to the reclaimed fibers. As drawbacks, it normally requires long reaction times (even several hours) combined with the use of strong acid solutions (e.g., nitric acid) and/or toxic solvents (e.g., benzyl alcohol and N,N-dimethylformamide (DMF)) and catalysts (homogeneous, in general; NaOH and KOH, as examples) to reach reasonably good yields of recovered fibers [15].

Liu et al. [16] used a nitric acid solution (8 M) at 90 °C to decompose the epoxy resin into low-molecular-weight compounds within 12 h, recovering the carbon fibers with low strength loss (around 1%). Lee et al. [17] also used a nitric acid solution (12 M) at 90 °C but in a circulating flow reactor (flowrate 1 cm/s) over 6 h, obtaining clean carbon fibers with a tensile strength loss of around 3%. Li et al. [18] studied the chemical recycling of carbon fiber/epoxy composites by oxidative degradation at 60 °C using acetone and H_2_O_2_; after 30 min of reaction, the decomposition ratio of the epoxy was above 90%, and CFs remained more than 95% of their original strength. Xu et al. [19] used a mixed solution of hydrogen peroxide and N,N-dimethylformamide at 90 °C for 30 min to recover carbon fibers with more than 95% of the tensile strength compared to virgin fibers. Wang et al. [20] used a combination of AlCl_3_/CH_3_COOH as a catalyst in the chemical recycling of carbon fiber reinforced polymers (CFRPs) by selective cleavage of a tertiary carbon–nitrogen bond. Under the optimal degradation conditions (catalyst mass fraction of 15 wt.%, 180 °C, 6 h), the recovery yield of the cured epoxy-resin matrix was around 97 wt.%, and the clean CFs recovered conserved about 98% of their original tensile strength. Lo et al. [21] used a catalytic oxidation method at 80 °C to recycle thermally stable benzoxazines. In their study, acetic acid, hydrogen peroxide, and a ruthenium catalyst were used in the depolymerization process, which lasted more than 24 h.

Evidently, the use of these harmful chemicals increases the environmental burden; therefore, there is a growing trend toward more environmentally benign solvents, such as water or alcohols, even though this may entail higher process temperatures. Such conditions often lead to a significant reduction in reaction times, which can help offset or even improve the overall efficiency of the treatment.

#### 1.2.2. High-Temperature Solvolysis

The use of solvents under high temperature and high pressure, mainly water or alcohols, has been more widely studied in the literature with the aim of developing effective recycling methods to obtain undamaged carbon fibers, with adequate surface and mechanical properties, short reaction times, and that avoid the use of harmful reagents [5,13,22,23,24]. This has led to growing interest in this option, which is the approach investigated in the present work. Depending on the temperature and pressure conditions, high-temperature solvolysis is normally carried out at near-critical or supercritical conditions of the solvent (or solvent mixture) used.

According to the literature, the use of water as a solvent is preferred over other solvents because it is environmentally friendly. Nevertheless, as it is shown below, many studies used solvents, such as ethanol, methanol, propanol, and acetone, and their mixtures with water. Since the critical point of alcohols (e.g., methanol T_c_ = 240 °C, P_c_ = 79.5 bar; ethanol T_c_ = 243 °C, P_c_ = 63.9 bar) is lower than that of water (T_c_ = 374 °C and P_c_ = 221 bar), the use of alcohols in CFRPs recycling would require less energy, but the use of water is greener and safer. In a circular-economy framework, an attractive scenario is the use of process-derived residues or wastewater streams as the solvent for these treatments.

Supercritical methanol has been used in the literature [25,26] to decompose the epoxy-resin matrix and, in general, recycling the CFRPs. At 270 °C and 80 bar for 90 min, the recovered carbon fibers had no heat damage.

Supercritical propanol has also been used by other authors [27,28,29,30,31], who reported that this process is an effective recycling technology for carbon fibers in epoxy-resin composites, yielding recycled fibers with mechanical properties comparable to those of virgin fibers. Yan et al. [30] found that with a temperature increase from 310 to 340 °C, the decomposition rate of the resin in the composites increased, but the tensile strength of the recycled fibers decreased from 3.7 to 3.0 GPa.

Sub- and supercritical acetones were found to be excellent for fast degradation of resin matrix [32]. For example, Okajima and Sako [33] reported that the decomposition efficiency increased with an increasing reaction pressure and acetone density, to a maximum value of 95.6% at 350 °C and 140 bar for 60 min, and the tensile strength reduction in recovered carbon fibers was insignificant. More recently, Vogiantzi et al. [23] carried out hydrothermal solvolysis of CFRPs using water or acetone at 300–400 °C, 85–300 bar, and 5–90 min, obtaining satisfactory results in terms of decomposition efficiency, but the treatment caused sizing loss in the CFs and a marked reduction of 18–38.6% in the Young’s modulus of the CFs.

As mentioned before, the use of near critical and supercritical waters as solvents is gaining attention progressively. On the one hand, subcritical water alone (without mixing with other solvents or without catalyst) cannot achieve the high decomposition efficiency of epoxy resins. On the other hand, supercritical water has high diffusivity, a high mass-transfer coefficient, low viscosity, and low dielectric constant, fostering fast hydrolysis reactions and good solubility of organic compounds; therefore, it is an excellent medium to effectively break down the polymer matrix without damaging the fibers.

Okajima et al. [34] studied the decomposition of epoxy resins and the recycling of carbon fibers by means of sub- and supercritical water. Clean carbon fibers were obtained from temperatures of 380 °C and pressures ≥ 250 bar. Piñero-Hernanz et al. [35] used a batch reactor without stirring at temperatures between 250 and 400 °C and at pressures between 40 and 280 bar, with reaction times of up to 30 min; a resin removal of 98 wt.% was achieved at the highest temperature. In another study, Bai et al. [36] used oxygen in supercritical water at 300 bar and 440 °C for 30 min and reached decomposition efficiency rates of 97%, while the mechanical and surface properties of the fibers were maintained. Kim et al. [37] removed up to 99.5% of the epoxy resin with supercritical water at 400 °C and 280 bar for 120 min of reaction in a 150 mL batch-type stirred reactor.

In order to propose an efficient method for carbon fibers recycling, while reducing the process temperature and/or the reaction time, the use of water must be combined with other solvent and/or with a catalyst. As it is described in some reviews [5,12,13], several studies also tested the use of solvent mixtures of water, alcohols and/or acetone [28,33,35,38] and the use of catalysts as KOH or ZnCl_2_ [32,39,40,41]. The positive effect of KOH addition has been clearly stablished in the literature; for example, Liu et al. [41] reported that the addition of small amount of KOH decreased the temperature from 275 °C to 210 °C for the complete degradation of epoxy resin when subcritical methanol was used as the solvent. Yan et al. [30], found that the addition of 1 wt.% of KOH improved the recovery efficiency of the composites significantly, but higher concentrations did not lead to further enhancement in the decomposition rate and the mechanical properties of the recycled fibers became poor. In a previous study by our group [42], similar hydrothermal treatments were conducted using KOH, NaOH and K_2_CO_3_ as catalysts under subcritical conditions (300 °C, ≈130 bar), achieving resin degradation rates up to 98% and fibers that retained more than 95% of their original tensile strength. Based on those findings, the present work explores whether similar degradation efficiencies can be achieved in the absence of alkaline catalysts by operating under more severe (subcritical to supercritical) conditions and using different solvent systems, and assesses the influence of ZnCl_2_ as an alternative homogeneous catalyst. The effect of ZnCl_2_ in high-temperature solvolysis has not been extensively studied. However, Keith et al. [38] reported that using an acetone/water mixture and ZnCl_2_ catalyst achieved a resin removal yield around 94% in 45 min at 300 °C, and the recovered fibers showed an increase in tensile strength after the process.

From the literature review, the use of water as a sole or major component of the reaction medium seems to be well established, and the use of an alcohol or acetone has been proposed as an option to reduce operating temperatures or reaction times. To our knowledge, the use of isopropanol (IPA) has not been widely tested by other authors and is justified by the fact that some residues from the semiconductor industry (wastewaters from the drying/cleaning stage of the electronic wafers manufacturing) contain great amounts of this alcohol [43,44]. In this work, IPA is used as a model solvent to evaluate the feasibility of employing industrial effluents as reaction media in hydrothermal recycling processes. Valorizing waste streams increase the circularity of the process. In this sense, our exploratory approach aims to assess whether alternative solvent systems, potentially derived from waste streams, can provide comparable degradation efficiencies under similar or milder operating conditions, or at least be applied without significantly compromising the effectiveness of carbon fiber recovery. The use of such residues as a source of IPA for CFRP recycling could represent a promising route towards improving the sustainability and feasibility of the reclamation process. Moreover, the addition of ZnCl_2_ may enhance the degradation rates of epoxy resins in high-temperature solvolysis processes. In this work, we study the effect of medium to high temperatures and pressures on the dissolution of the composite polymer matrix and the recovery of carbon fibers, using water, isopropanol, water/isopropanol, or water/acetone as solvents under subcritical and supercritical conditions, with and without ZnCl_2_ as a homogeneous catalyst.

## 2. Materials and Methods

### 2.1. Composite Off-Cuts Specimens, Solvents, and Catalyzers

**Figure 2:**
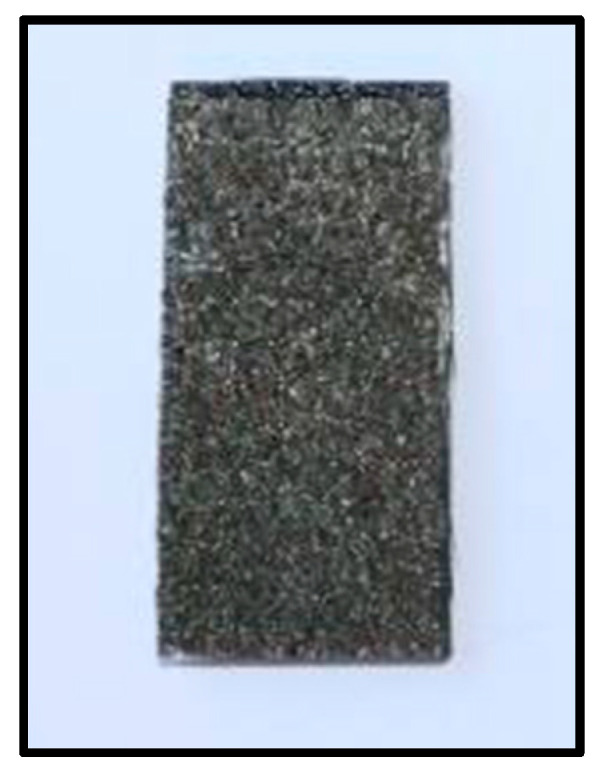
Picture of an original composite off-cut.

Carbon fiber-reinforced epoxy unidirectional tape flat composite off-cuts were used as feedstock (see Figure 2). All the specimens were aeronautical-grade unidirectional tape composites (i.e., manufactured by stacking pre-preg sheets with CFs oriented in the same direction), which were kindly provided by TITANIA Ltd. (Cádiz, Spain). Two different types of composite off-cuts were used in this work, using their basic specifications, as shown in Table 1.

The Milli-Q deionized water was produced on site. The isopropanol (IPA; C_3_H_8_O) at 100% purity was supplied by BRENNTAG (Sevilla, Spain). The acetone (C_3_H_6_O) at 99.5% purity and the zinc chloride (ZnCl_2_; purity ≥ 98%) were supplied by SIGMA-ALDRICH (St. Louis, MO, USA). Finally, high-purity nitrogen and the pure helium were supplied by CARBUROS METÁLICOS (Barcelona, Spain) in 200 bar bottles.

### 2.2. System and Procedure

**Figure 3:**
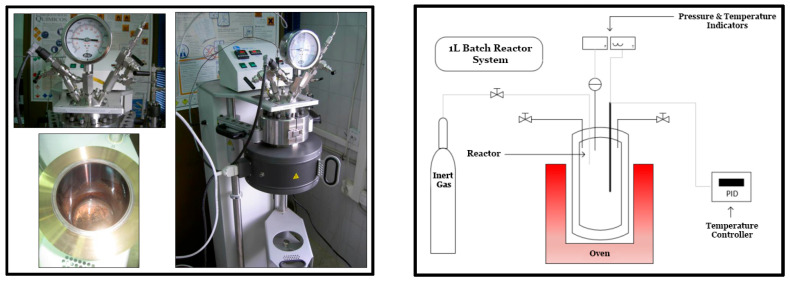
Picture and schematic diagram of the batch reactor used to perform the hydrothermal treatments at laboratory scale.

The hydrothermal treatments of the composite off-cuts were performed in a 1 L PARR (Parr Instrument Company, Moline, IL, USA) 4570 Series 316 stainless-steel unstirred batch reactor (Figure 3), which can operate at a maximum temperature of 570 °C (accuracy of ±3 °C, using a 2300 W PID-controlled oven) and at pressures up to 277 bar (a). Prior to each hydrothermal test, to ensure the absence of oxygen during the experiments, the reactor was gently purged with either nitrogen (N_2_) or helium (He) from pressurized cylinders for 15 min. When needed, the initial reactor pressure required to reach the final testing pressure was set using N_2_ or He from the same cylinders.

### 2.3. Analytical Methods

#### 2.3.1. Thermogravimetric Analysis

Thermogravimetric analysis (TGA) of the original composite was performed using a PERKINELMER TGA-7 instrument (PerkinElmer, Waltham, MA, USA) to characterize its thermal decomposition behavior.

#### 2.3.2. Decomposition Rate

The decomposition rate (*DR*) of the composite specimens, expressed as the percentage of epoxy-resin matrix degraded in each hydrothermal run, was determined by gravimetric measurements using a SARTORIUS digital precision balance. *DR* was calculated according to Equation (1) [45], as follows:

(1) $$DR\left(\%\right)=\frac{M_{c}-M_{r}}{M_{e}}\times 100$$

where *M_c_* is the mass of the original composite specimen, *M_r_* is the mass of the treated composite specimen, and *M_e_* is the mass of the epoxy-resin matrix within the original composite specimen. Prior to weighting, all samples were dried in a laboratory oven at 105 °C for 24 h.

#### 2.3.3. Scanning Electron Microscopy

The surface morphology and diameter of the carbon fibers before and after the hydrothermal treatment were examined by scanning electron microscopy (SEM) using a NOVA NANOSEM 450 (FEI Company, Hillsboro, OR, USA) through the acquisition and analysis of the corresponding micrographs.

#### 2.3.4. Single-Fiber Tensile Tests

Single-fiber tensile tests (SFTTs) were carried out by TITANIA Ltd. (Cádiz, Spain), in accordance with the ISO 11566 standard [46] to evaluate the tensile strength and elastic modulus of the recycled carbon fibers.

#### 2.3.5. Gaseous Effluent Analysis

The composition of the gaseous effluent (H_2_, CO_2_, O_2_, N_2_, CH_4_, and CO) was determined using an HP 6890 PLUS gas chromatograph with a thermal conductivity detector (TCD). Two columns coupled in series were used to separate CO from CO_2_: a PORAPAK-Q column followed by a CARBOSIEVE carbon molecular-sieve column (SUPELCO).

Gas production is expressed as the gas yield (mol/kg), using the following Equation (2), which is defined as the number of moles of a specific component in the reactor gas phase divided by the initial mass of organic matter in the experiment, which can be the epoxy resin in the composite sample and the IPA or acetone in the solvent when applied [47]:

(2) Gas Yield molkg=Gas moles (mol)Initial weight of organic matter (kg)

The moles of each gas were obtained using the ideal gas law under the sampling conditions in combination with the measured gas composition. This approach was applied after cooling and depressurizing so that deviations from ideal behavior at reaction temperature/pressure did not affect the calculation of the moles in the gas phase.

#### 2.3.6. Liquid Effluent Analyses

The liquid effluent was analyzed for pH, conductivity, chemical oxygen demand (COD) and total organic carbon (TOC). All the analyses were performed according to “*Standard Methods for the Examination of Water and Wastewater*” [48]. COD analysis was conducted by closed reflux colorimetric method (5220D). The combustion-infrared method (5310B) was conducted in a TOC analyzer SHIMADZU 5050 (Shimadzu Corporation, Kyoto, Japan).

## 3. Results

### 3.1. Thermogravimetric Behavior of the Original Composite Specimen (SpA)

**Figure 4:**
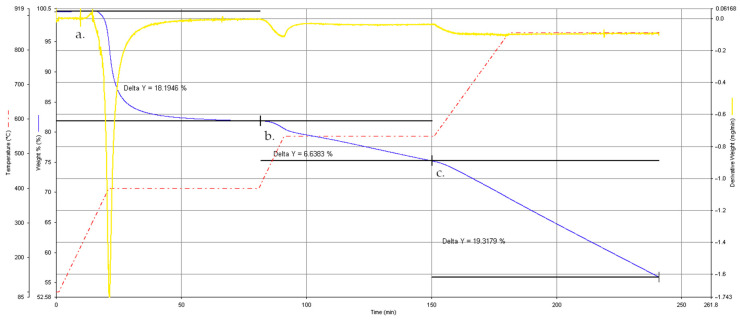
Thermogravimetric and derivative mass–loss (DTG) curves of the original composite specimen (SpA). Peaks (a), (b), and (c) correspond to the main decomposition stages of the epoxy matrix.

To assess the thermal stability of the composite material used in this work, thermogravimetric analysis (TGA) was performed on an original SpA specimen. Figure 4 displays the mass–loss curve (thin solid blue line), the derivative mass–loss (DTG) curve (thick solid yellow line), and the temperature ramp (red dashed line).

The sample remained thermally stable up to approximately 350 °C, showing no significant mass change. A sharp weight loss was then observed between 380 °C and 430 °C (a), corresponding to the onset of epoxy matrix decomposition. A second, broader event (b) occurred at around 550 °C, associated with the progressive degradation of the remaining resin and the partial decomposition of the fiber sizing layer [49,50]. Finally, a third stage (c) appeared beyond 600 °C, where a continuous weight loss was recorded, indicating the gradual degradation of residual organic matter and, to some extent, of the carbon fibers themselves.

In summary, the composite’s high thermal stability makes it difficult to achieve meaningful and selective matrix removal by conventional thermal methods without harsh conditions, thereby supporting the hydrothermal approach investigated in this work to promote selective epoxy decomposition under milder conditions while preserving carbon fibers and, where possible, their sizing layer.

### 3.2. Experimental Results Overview

The present study comprised a total of 23 experiments, the objective of which was to investigate the impact of different operating conditions on carbon fiber recovery. The results of these experiments are set out in Table 2.

Various solvents were evaluated, as follows: water, isopropanol, and acetone. The latter two were also used as aqueous solutions with different mass concentrations. For these solvents, tests were conducted over a broad range of pressures (P) and temperatures (T), spanning from 30 to 250 bar and from 200 to 550 °C, respectively. Of particular interest was whether the solvents under these conditions were in the supercritical state (SC). The table employs “Y” (yes) to denote a solvent in supercritical state and “N” (no) if it is below its critical point. Water has its critical point at 374 °C and 220.6 bar, acetone at 235 °C and 48 bar, and IPA at 238 °C and 47.6 bar. For aqueous solutions, the critical point depends on composition and typically lies between the critical points of the pure components. The effect of increasing the system’s initial pressure (P_0_) through the addition of an inert gas, such as nitrogen (N_2_), has also been evaluated.

Most experiments were conducted using SpA composite off-cuts samples; however, some SpB composite off-cuts samples were also tested (see Section 2 (Materials and Methods) for a description of the samples). The reaction time of the tests (t_r_) and the solvent-to-solid ratio were other variables studied, with the ratio defined as the volume of solvent in milliliters per mass of the composite sample in grams. Finally, some tests were conducted using ZnCl_2_ as a catalyst.

It should be noted at the outset that six of the experiments exceeded a decomposition rate of 80%, all of them performed under supercritical solvent conditions. Of particular interest are experiments LC4, which achieved a decomposition rate of close to 90% without a catalyst, and LC23, which utilized ZnCl_2_ as catalyst and achieved a rate of 92%.

To assess the reproducibility of the process, tests LC10 and LC11—performed under identical operating conditions—were analyzed. The resin decomposition rates (*DRs*) obtained were 45% and 47%, respectively, which corresponds to a difference of 2% and, therefore, to a relative error of approximately 4%. This value was taken as a reference for the experimental uncertainty associated with the rest of the tests.

It has also been observed that the type of composite had a limited effect on resin degradation under the tested conditions. Using SpA (LC16) and SpB (LC17) at 400 °C with IPA resulted in *DR* values of 83% and 80%, respectively, suggesting that the matrix composition plays a minor role under supercritical conditions.

### 3.3. Effect of Reaction Time on Resin Degradation

**Figure 5:**
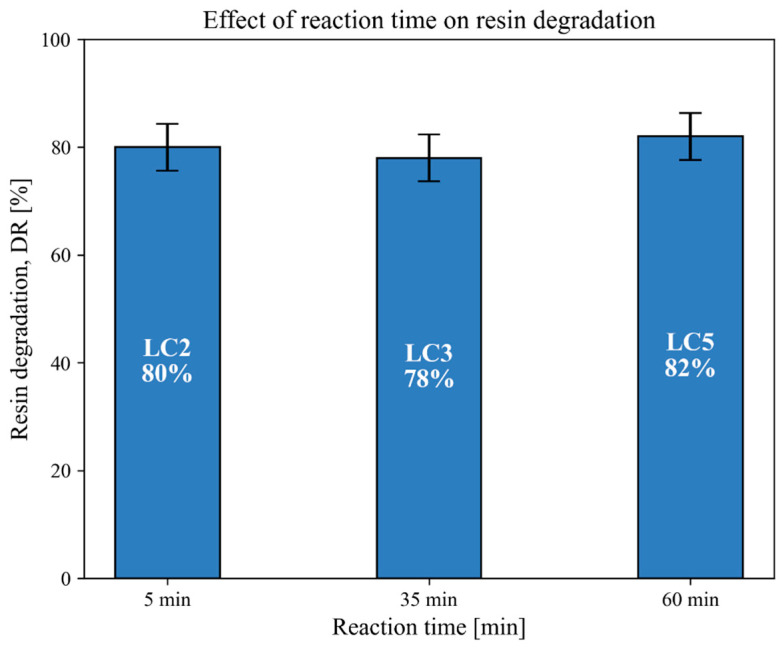
Decomposition rates as a function of reaction time using water as the solvent at 400 °C, 230–240 bar, and 165–175 mL solvent/g composite, with SpA as the composite material.

To investigate the influence of reaction time on the resin degradation rates (*DRs*) obtained through the hydrothermal process, three tests were performed with varying durations, as follows: 5 min (LC2), 35 min (LC3), and 60 min (LC5). The rest of the operating conditions were kept constant, as follows: temperature of 400 °C, initial pressure between 8 and 11 bar, pure water as the solvent, no catalyst added, and a solvent-to-composite mass ratio of around 170 mL/g. The resulting *DR* values (%) are presented in Figure 5.

Although an increase in reaction time from 5 to 60 min might be expected to improve resin decomposition, the results obtained under supercritical conditions using water as the solvent show only a minor variation in *DR* (80–82%). These results indicate that, under supercritical water conditions, increasing the reaction time beyond 5 min does not lead to a significant improvement in resin removal efficiency.

This behavior contrasts with what was observed under subcritical conditions in a previous study by the authors [42], where longer reaction times resulted in substantially higher degradation rates—from 51% at 35 min to 98% at 120 min—when using a 0.1 M KOH catalyst and a 5 wt.% water–IPA solution at 300 °C. Similar trends under subcritical conditions have also been reported in the literature, such as by Yuyan et al. [51], who observed a strong correlation between reaction time and resin degradation efficiency at 260 °C.

### 3.4. Interrelated Effects of Pressure and Solvent Ratio on Resin Degradation

**Figure 6:**
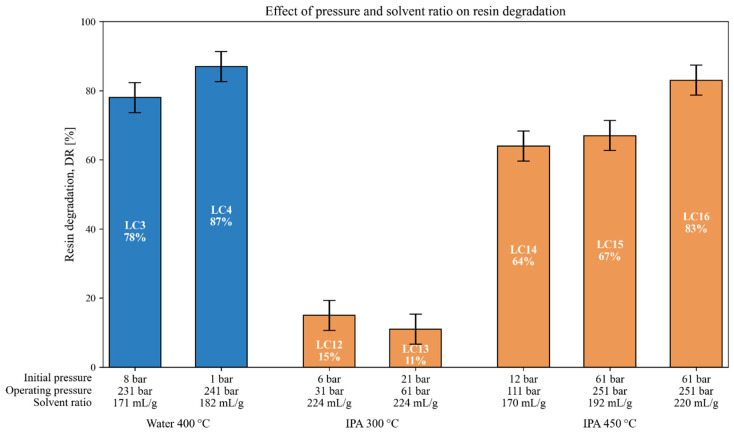
Resin degradation rates using water or IPA at different temperatures, with SpA as composite. Each test combines variations in initial pressure, operating pressure, and solvent ratio.

In this section, the combined influence of three interrelated operating variables—initial pressure (P_0_), operating pressure (P), and the solvent-to-composite ratio—is analyzed, as these parameters are often modified simultaneously due to experimental constraints. This is particularly true when trying to reach specific final conditions within a batch reactor. For instance, under supercritical conditions, a given final pressure can be achieved by increasing either the initial N_2_ pressure or the solvent volume; however, increasing the solvent volume inevitably raises the solvent to composite ratio. As a result, these three variables are naturally coupled in many of the experiments carried out, which makes it difficult to isolate their individual effects and thus requires a joint interpretation. The resulting *DR* values (%) are presented in Figure 6.

A representative example of this interaction can be seen in tests LC3 and LC4, both performed with water at 400 °C for 35 min using the same composite. Reducing the initial nitrogen pressure from 8 to 1 bar and slightly increasing the solvent to composite ratio from 171 to 182 mL/g led to an increase in *DR* from 78% to 87%. Although a small increase in the final pressure was also observed (from 231 to 241 bar), the most likely contributors to this improvement are the lower initial inert-gas pressurization (i.e., lower P_0_), which may reduce dilution of the reactive solvent phase and/or the higher solvent volume. This observation aligns with the hypothesis raised during the experimental design that high initial N_2_ pressure, particularly under supercritical conditions, might dilute the solvent phase and reduce its solvent strength. In this context, tests such as LC4 and LC8 (without initial pressurization) achieved the highest *DRs* within their respective solvent groups (water and water–IPA mixtures).

A more marked trend was observed in the IPA-based tests at 450 °C using the same composite (LC14–LC16). In this group, P_0_ increased from 12 to 61 bar, the solvent ratio from 170 to 220 mL/g, and the operating pressure from 111 to 251 bar. The *DR* improved accordingly, from 64% (LC14) to 67% (LC15) and 83% (LC16). Since all three variables increased simultaneously, their individual contributions cannot be decoupled. However, the stepwise progression suggests that both solvent loading (ratio) and operating pressure have a positive influence under these conditions.

Conversely, LC12 and LC13, performed at 300 °C with pure IPA and the same solvent ratio (224 mL/g), show only minimal variation in *DR* (15% vs. 11%) despite a significant difference in P_0_ (6 vs. 21 bar) and in the final pressure (31 vs. 61 bar). These results suggest that, under subcritical conditions and in the absence of a catalyst, the effects of P_0_ and P are less relevant or at least less dominant than solvent chemistry and temperature.

The role of the solvent to composite ratio has also been discussed in a previous study by the authors of [42], in which this parameter was varied from 223 to 448 mL/g under subcritical conditions (300 °C, 130 bar) using a 0.1 M KOH catalyst and a 5 wt.% IPA solution. In that case, the *DR* increased only slightly from 90% to 95%, with no clear benefit observed beyond ≈300 mL/g. Similarly, Sokoli et al. [32] found that, for a hybrid composite treated under near-critical water conditions, degradation remained consistently high (≈95%) for solvent ratios between 0.8 and 1.96 mL/g. These findings suggest that, once a certain solvent excess is reached, further increases in the solvent ratio have only marginal effects.

Overall, while the solvent ratio and operating pressure often correlate with improved degradation, the isolated effect of P_0_ remains ambiguous and potentially system dependent. Additional experiments with a more controlled variation of each parameter would be required to establish their individual impact with greater confidence.

### 3.5. Effect of IPA Content in Solvent Mixture on Resin Degradation

**Figure 7:**
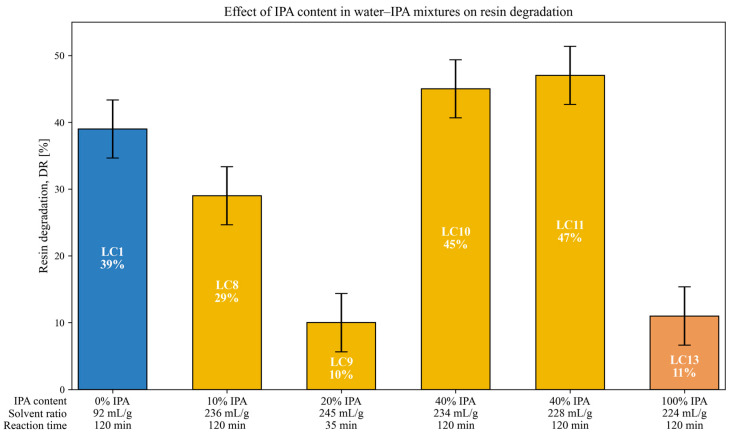
Resin degradation rates for different IPA concentrations in water–IPA mixtures, with SpA as the composite material. Each bar corresponds to a specific test, showing the IPA content (%), solvent to composite ratio (mL/g), and reaction time (min).

The influence of the isopropanol (IPA) content in water–IPA mixtures on resin degradation (*DR*) was explored through a series of tests with varying IPA concentrations: 0% (LC1), 10% (LC8), 20% (LC9), 40% (LC10 and LC11), and 100% (LC13). Despite certain variations in operating conditions, several patterns and observations are worth discussing. Figure 7 summarizes the results of these tests.

LC1, conducted with pure water at 300 °C for 120 min, resulted in a *DR* of 39%, which is relatively high within this group, especially considering that it was obtained with a low solvent to composite ratio (92 mL/g), which has been previously associated with reduced degradation efficiency.

In contrast, LC8 and LC10, carried out under similar conditions but with 10% and 40% IPA respectively and higher solvent ratios (≈235 mL/g), yielded *DR* values of 29% and 45%. These results suggest a possible beneficial effect at 40% IPA. However, LC9 (20% IPA) produced a notably low *DR* (10%), which can likely be attributed to its significantly shorter reaction time (35 min), as observed in a previous study [42], where reaction time significantly affected degradation efficiency under subcritical conditions.

LC13, performed with pure IPA, also resulted in a low *DR* (11%). Interestingly, this value is lower than that obtained with pure water under subcritical conditions (LC1, 39%), highlighting the better performance of water in this case, despite both tests being conducted under similar temperature and pressure conditions. Under these conditions, the IPA in LC13 is in a supercritical state, while water in LC1 remains subcritical. This outcome may reflect the lower efficiency of IPA as a solvent compared to water. This interpretation is further supported by the comparison between LC4 (pure water) and LC7 (5% IPA), both conducted under similar supercritical conditions, where better degradation was observed with water. Although these two tests involved different composite materials, the influence of the composite types used in this study was shown to be limited under supercritical conditions, as discussed earlier in this work.

**Figure 8:**
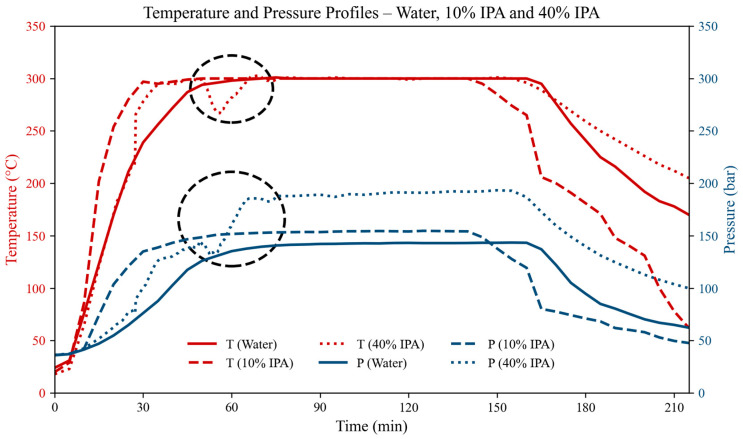
Temperature and pressure evolution during LC1 (Water), LC8 (10% IPA), and LC10 (40% IPA). The marked regions highlight the area where a temperature drop and pressure increase were observed, indicating a possible marked endothermic behavior and phase transition.

Among the tests considered, LC10 and LC11 stand out for their relatively high *DR* values at 40% IPA. Both were carried out at 300 °C for 120 min and, unlike the other tests, showed a distinctive temperature and pressure profile (Figure 8). In both cases, a sudden drop in temperature was observed during the reaction, indicative of a strong endothermic event, followed by a marked increase in pressure. This behavior could be associated to a phase transition in the binary solvent system and may help explain the enhanced degradation observed in these tests. According to data reported by Anikeev et al. [52], the critical point for a 40% IPA solution lies around 280 °C and 100 bar (conditions close to those reached in LC10 and LC11). Thus, there were indications that operating in close proximity to the critical region could enhance the solvent’s reactivity and contribute to the comparatively higher *DR* values obtained. Two important notes should be considered when seeking an explanation for this phenomenon: (i) we performed six replicated tests under these conditions, and the same behavior was observed in all cases, confirming the reproducibility of the event; (ii) all of these tests were conducted with an initial N_2_ pressure of 37 bar in addition to the binary solvent mixture.

Although this phenomenon is not the main focus of the present study, we considered it worthwhile to report. Nevertheless, these observations should be regarded as preliminary, and a dedicated experimental campaign will be required to elucidate the underlying mechanism and assess its relevance for process optimization.

**Figure 9:**
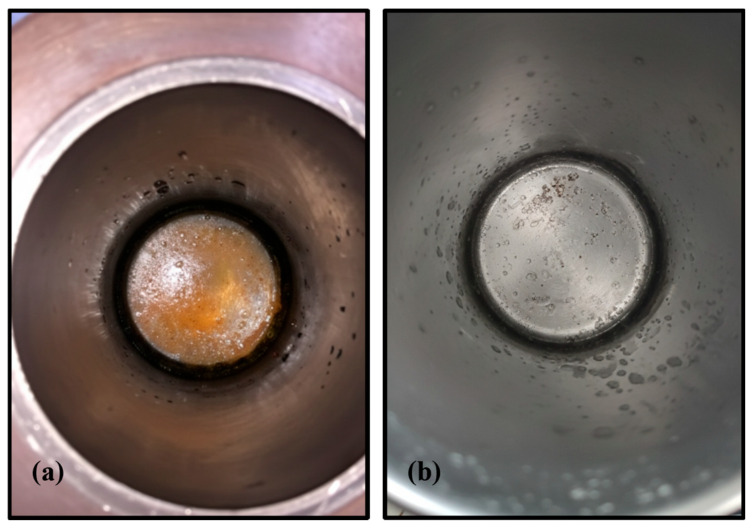
Photographs of the reactor interior after treatments using (**a**) water (LC4) and (**b**) IPA (LC7) as solvents.

In addition to the degradation performance, the use of IPA also presented operational advantages. At high temperatures (400–550 °C), tests using IPA as solvent—such as LC7—resulted in significantly cleaner internal reactor surfaces compared to those where water was used, such as LC4. As shown in Figure 9, degraded resin residues were visibly deposited on the base and walls of the reactor after water-based treatments, whereas IPA-based operations left the surfaces noticeably cleaner. This represents a practical advantage in terms of reactor maintenance and process scale-up.

In summary, while a clear quantitative relationship between IPA concentration and resin degradation cannot be established based on the current data, several patterns support further exploration. In particular, the behavior of the 40% IPA mixture appears to be linked to a near-critical transition of the solvent system, which could play a key role in enhancing decomposition under certain conditions. Additional tests under controlled conditions would help to clarify the effect of IPA concentration in hydrothermal treatments.

### 3.6. Effect of Temperature, Operating Regime, and Catalyst on Resin Degradation

**Figure 10:**
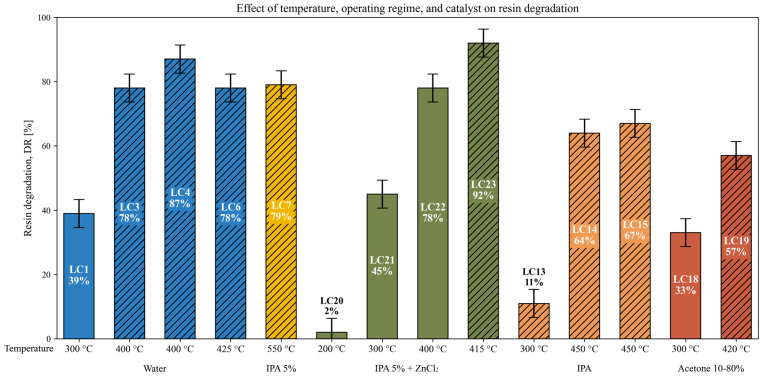
Resin degradation as a function of reaction temperature, operating regime (subcritical or supercritical), and catalyst presence. Bars are grouped by solvent type and ordered by increasing temperature. Supercritical conditions are indicated with diagonal hatching.

The results presented in Figure 10 illustrate the influence of reaction temperature, operating regime (subcritical or supercritical), and the presence of a catalyst on the degradation of the resin matrix for different solvents and solvent mixtures. In general, increasing the reaction temperature leads to higher resin degradation (*DR* %) values; however, once supercritical conditions are reached, further temperature increases do not result in additional improvements in degradation. This is reflected in test LC7, where even at 550 °C, a *DR* of only 79% is achieved. Supercritical conditions are indicated with diagonal hatching.

At 300 °C, the degradation efficiency varies considerably depending on the solvent used. Water (LC1) led to a *DR* of 39%, whereas pure IPA (LC13) under the same conditions achieved only 11%, suggesting again that water is more effective than IPA as solvent. As discussed in the previous section, tests with low IPA concentrations—such as LC8 (10% IPA)—also show a moderate decrease in resin degradation efficiency (*DR* = 29%). Therefore, it can be assumed that using 5% IPA without catalyst, a concentration selected to simulate the composition of organic wastewaters such as those generated during semiconductor cleaning and drying processes, as well as to benefit from its reactor–cleaning effect, would also result in a lower *DR* %, despite being more similar to water than to pure IPA. However, test LC21, which used 5% IPA with ZnCl_2_ as catalyst, achieved a *DR* of 45%, suggesting that the catalyst helps compensate for the loss of efficiency associated with the presence of IPA in the solvent.

In our previous work [42], the use of KOH as a catalyst enabled effective resin degradation at 300 °C, with significantly higher *DR* values than those obtained here under similar thermal conditions using ZnCl_2_ (e.g., LC21, *DR* = 45%).

The limited degradation observed in LC20 (5% IPA + ZnCl_2_, 200 °C, *DR* = 2%) reinforces the need for a minimum thermal input to activate the process, regardless of the solvent system or the presence of a catalyst.

At higher temperatures (400–415 °C), similar patterns are observed. Water-based systems (LC3–LC4) yielded *DR* values between 78 and 87%, comparable to LC22 (IPA 5% + ZnCl_2_, 78%). In contrast, using pure IPA (LC14–LC15) resulted in lower *DR* values, between 64 and 67%. However, a further increase in *DR* was observed when using the catalyst at slightly higher temperature, reaching 92% *DR* (LC23, 415 °C).

Taken together, these results suggest that both the operating regime and, to a lesser extent, the catalyst can contribute to enhancing the hydrothermal degradation of epoxy matrices.

**Figure 11:**
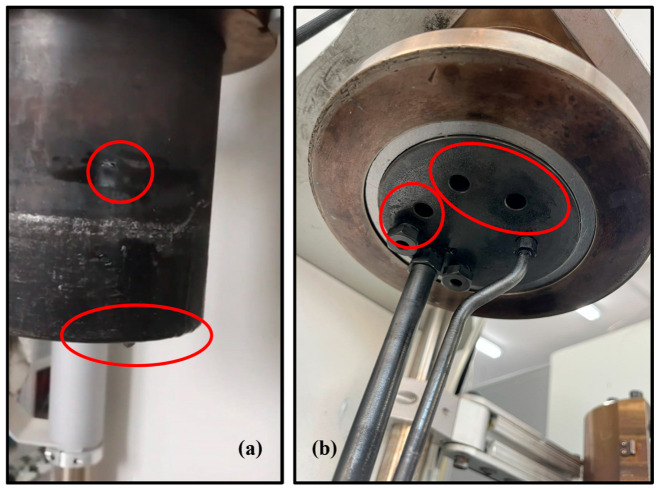
Macroscopic corrosion damage observed (marked with red circles) after experiments using ZnCl_2_ as the catalyst, particularly under supercritical water conditions: (**a**) severe pitting corrosion on the walls of the 316 stainless-steel reactor vessel; (**b**) extensive surface pickling on the reactor head assembly.

It should be strongly emphasized that multiple signs of severe pitting corrosion were observed on the 316 stainless-steel reactor vessel walls (Figure 11a), together with pronounced surface pickling on the head of the unit (Figure 11b) when ZnCl_2_ was used, particularly under supercritical water conditions; these damage features were readily discernible by visual inspection, without the need for microscopic analyses, indicating that this catalyst is highly aggressive toward the reactor materials under the investigated conditions.

The use of ZnCl_2_ in this study was motivated by previously reported promising results obtained in the literature under hydrothermal conditions [38]. However, regarding our present work, due to the corrosion issues observed and the only moderate performance improvement achieved by using ZnCl_2_, its use in future studies and its scale-up are not recommended. These findings should not discourage further investigation of alternative catalysts, such as KOH, which we employed successfully in our previous work [42].

Additionally, tests using acetone–water mixtures (LC18 and LC19) yielded intermediate resin degradation values (33% and 57%, respectively), suggesting that acetone may offer limited performance under these conditions compared to water or IPA-based systems.

### 3.7. Scanning Electron Microscopy (SEM)

The morphology and surface condition of the carbon fibers recovered through the hydrothermal treatment were examined by scanning electron microscopy (SEM). The analysis focused on identifying potential structural damage, resin residues, and changes in the sizing layer. To this end, fiber diameters were measured and the sizing thickness was estimated, as preserving this coating is essential for the potential direct reuse of the fibers in manufacturing new composite materials or other goods.

The sizing is a thin and uniform epoxy layer applied to the surface of carbon fibers during their manufacturing to protect them from mechanical damage during handling and processing. Beyond this protective function, it also enhances the chemical compatibility and mechanical performance of the fibers and plays a critical role in promoting adhesion between the fibers and the surrounding resin matrix [53].

**Figure 12:**
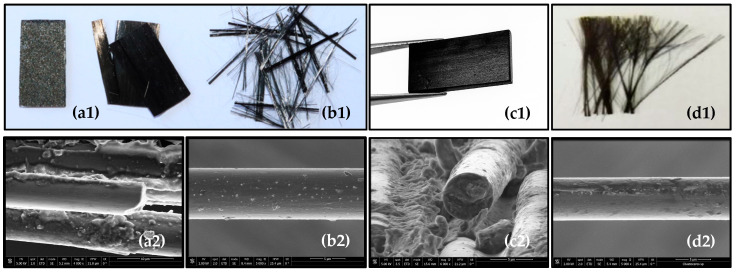
Representative photographs (**a1**–**d1**) and SEM (**a2**–**d2**) images of the composite material before and after hydrothermal treatment under different conditions. The original type SpA composite sample is shown in (**a1**,**a2**) and the original type SpB is shown in (**c1**,**c2**), while the recovered fibers correspond to tests LC4 (**b1**,**b2**) and LC23 (**d1**,**d2**).

The SEM images of the original composite samples of types SpA and SpB (Figure 12a,c) show the carbon fibers embedded within the epoxy matrix. Figure 12b,d display the fibers recovered from tests LC4 (400 °C; 241 bar; 35 min; pure water; no catalyst; SpA) and LC23 (415 °C; 233 bar; 120 min; 5 wt.% IPA; 0.1 M ZnCl_2_; SpB), respectively. In both cases, the fibers exhibit clean surfaces with no significant resin residues, indicating highly efficient matrix removal, consistent with the *DR* values of 87% and 92% achieved in these tests. The photographs of the recovered fibers (b1 and d1) show that, in both cases, the fibers are fully separated from the matrix.

To assess the preservation of the sizing layer, the diameters of both the original and recovered fibers were measured. The difference between the average diameter of the original fibers and the nominal diameter specified by the manufacturer (7.1 μm for SpA and 5 μm for SpB) was used to estimate the sizing thickness, as shown in Table 3. In addition, the diameters of the recovered fibers were used to evaluate whether the sizing layer was largely preserved after treatment.

The results presented in Table 3 show that the average diameters of the recovered fibers are very similar to those of the original materials. In both cases (SpA and SpB), no significant reduction in fiber diameter was observed after treatment, indicating that the carbon fibers were not degraded by the hydrothermal process. The estimated sizing thicknesses are also consistent with those of the original samples, suggesting that the applied conditions were sufficiently mild to preserve the sizing coating and the fiber surface.

### 3.8. Tensile Strength Assays

**Figure 13:**
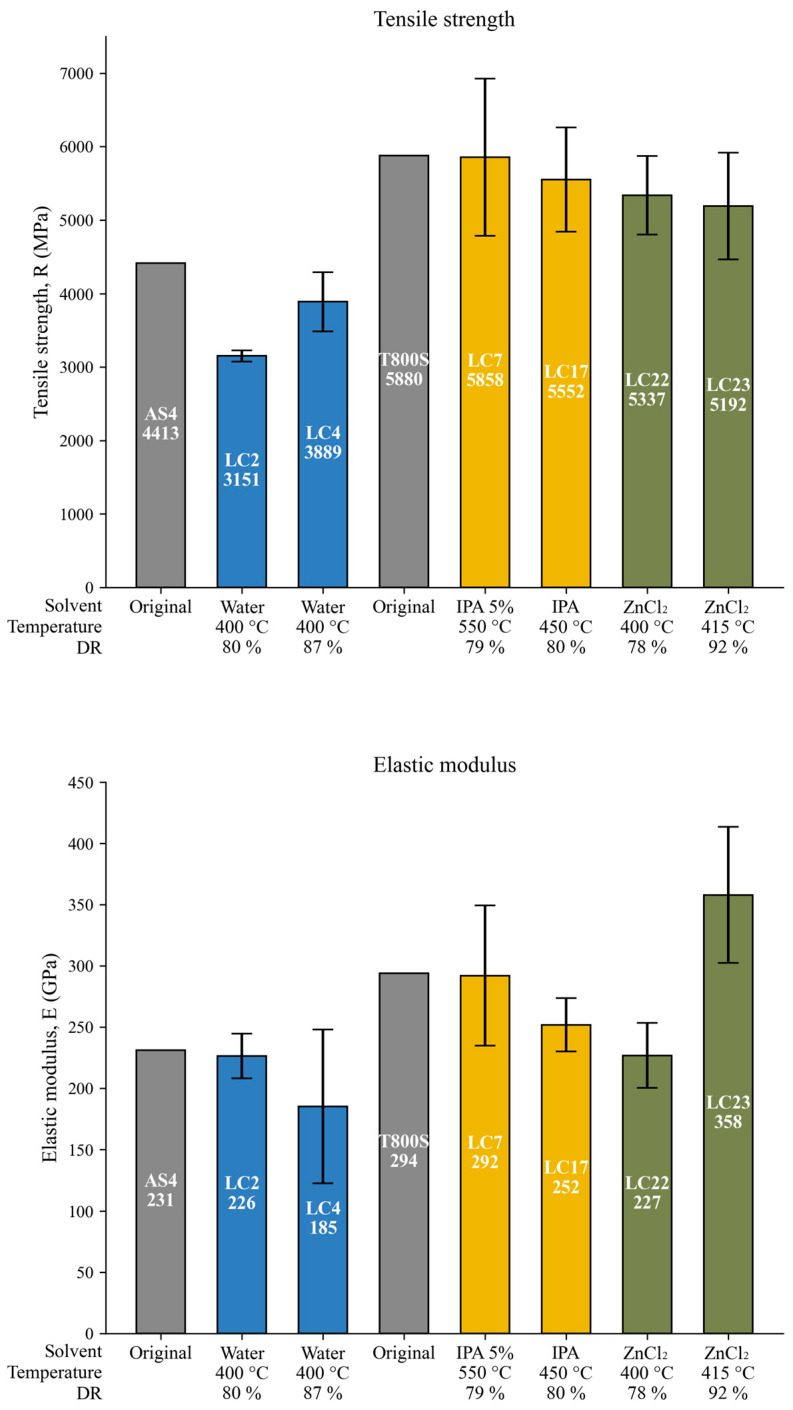
Tensile strength (*TS*) and elastic modulus (*E*) of the original and recovered carbon fibers.

Single-fiber tensile tests (SFTTs) were carried out to evaluate the tensile strength (*TS*) and elastic modulus (*E*) of the recovered carbon fibers, selected to cover a broad range of hydrothermal conditions, including the most severe ones. These tests allow for the assessment of the preservation of the mechanical performance of the fibers after matrix removal. The results are presented in Figure 13, which compares the *TS* and *E* values of the recovered fibers with those of the original materials. In this work, AS4 and T800S fibers were the reinforcing materials used in composite types A (SpA) and B (SpB), respectively.

The figure shows the mechanical properties of the untreated original fibers and those of recovered fibers obtained under different hydrothermal conditions. Internal labels indicate the test name and the corresponding average value, while error bars represent the confidence intervals of the measurements. In the case of tests LC22 and LC23, the label “ZnCl_2_” refers to the presence of zinc chloride as a catalyst in a 5% IPA solution, although the complete solvent composition is not specified in the figure for clarity and visual consistency.

As shown in Figure 13, the *TS* of the recovered fibers remains close to the original values in most cases. For the AS4 fiber samples, test LC4 reached a *TS* of 3889 MPa, corresponding to a moderate reduction of 12% compared to the original (4413 MPa). In contrast, LC2 presents a more significant decrease (3151 MPa, 29% reduction). For the T800S fiber samples, the *TS* values are remarkably well preserved under all conditions, particularly in LC7 (5858 MPa, 0.4% reduction) and LC17 (5552 MPa, 5.6% reduction), both using IPA-based solvents. Even the samples treated in the presence of ZnCl_2_ (LC22 and LC23) maintained high strength retention (5337 and 5192 MPa, with 9.2% and 11.2% reductions, respectively).

The elastic modulus shows trends consistent with tensile strength and is generally well preserved. The lowest *E* value was measured in LC4 (185 GPa), representing a 19.8% reduction. In contrast, LC23 displays an unusually high modulus (358 GPa), exceeding the nominal value of the original T800S fibers by 21.7%. While such increases in *E* are typically associated with high-temperature graphitization (>2500 °C) [54], other studies have shown that certain metal-catalyzed processes can promote partial graphitic ordering at much lower temperatures (≈900–1000 °C), particularly in the presence of metal salts (Li, Ca, Ni) [55].

Although LC23 was processed at only 415 °C, the combination of ZnCl_2_ as a catalyst, an IPA–water solution as solvent, and supercritical high-pressure conditions may have facilitated local microstructural rearrangements. In fact, pressure-enhanced orientation and crystallinity have been observed in carbonaceous fibers treated under supercritical conditions [56]. Therefore, the elevated *E* in LC23 could be explained by the synergistic effect of these chemical and physical factors, rather than by a measurement artefact.

These findings are consistent with those reported in our previous work [42], where fibers treated in supercritical water (test P11) exhibited a significant reduction in elastic modulus (22.4%), similar to the behavior observed here in LC4. In contrast, the preservation of both TS and E observed in tests using IPA–water solutions, with or without ZnCl_2_ (e.g., LC7, LC17, LC22, and LC23), aligns with the results of test P12 (2.5% *TS* reduction and 4.8% *E* reduction), which also employed a 5% IPA solution with a different catalyst (KOH 0.1 M). This parallel supports the observed trends and underscores the potential detrimental effect of water under supercritical conditions.

The hydrothermal recycling conditions applied in this study were proven effective in recovering carbon fibers with mechanical properties close to those of the original materials, particularly when suitable temperature–solvent combinations are employed.

### 3.9. Gaseous Effluent

**Figure 14:**
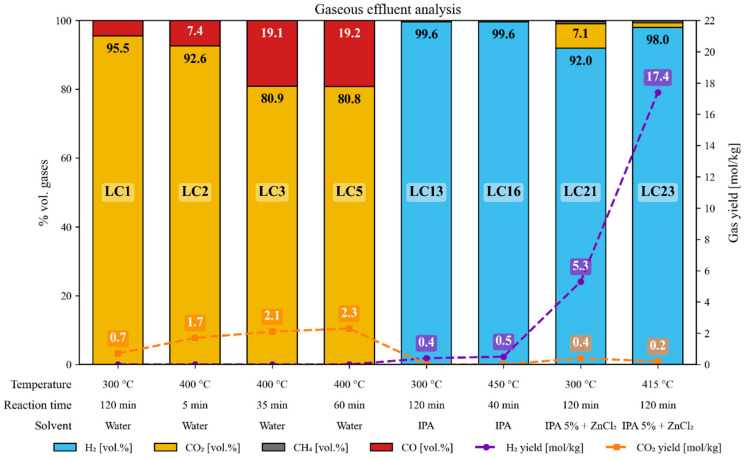
Gas composition and hydrogen and carbon dioxide yields for the gaseous effluents produced during the hydrothermal treatments of the composite specimens.

During the hydrothermal treatments aimed at recovering carbon fibers, a gaseous fraction was generated and subsequently characterized to determine its composition. Figure 14 summarizes, for a selection of representative tests, the volumetric composition of the gaseous phase and the corresponding specific gas yields.

In the tests conducted with water as the reaction medium (LC1–LC5), the gas phase consisted mainly of CO_2_ (≈81–96 vol.%), with minor fractions of CO (≈4–19 vol.%), and without the formation of energy-rich gases such as H_2_ or CH_4_. The specific CO_2_ yields were low but increased slightly with higher temperature and longer reaction time, together with an increase in the CO fraction. Under these conditions, the only organic source is the epoxy matrix, which progressively dissolves into the aqueous phase during the hydrothermal treatment.

In contrast, the experiments carried out with pure isopropanol (LC13–LC16) showed a drastic change in gas composition, being dominated almost entirely by H_2_. This behavior suggests the occurrence of isopropanol reforming reactions, favored by the higher temperature (≥400 °C) and the reducing nature of the solvent, which promotes hydrogen release and C–O bond cleavage.

When ZnCl_2_ was used as a catalytic additive together with small amounts of isopropanol (LC21 and LC23), the hydrogen yield increased markedly (reaching ≈ 17 mol H_2_/kg), while the gas remained highly pure (≈92–98 vol.% H_2_). This observation supports the catalytic role of ZnCl_2_ in promoting dehydrogenation and reforming reactions of the isopropanol present in the solvent. The high selectivity toward H_2_, together with the low CO_2_ content, indicates that under these conditions, the hydrothermal environment favors hydrogen transfer and redox pathways over simple thermal decomposition of the organic components.

Overall, these results show that the solvent and, when applicable, the catalyst govern both the composition and the amount of gas produced during the process. The formation of H_2_-rich streams under specific conditions further suggests their energetic valorization as a byproduct, a point that is particularly relevant for scaling up the hydrothermal carbon-fiber recycling process.

### 3.10. Liquid Effluent

The analysis of the liquid effluents completes the study of all of the streams generated during the hydrothermal recycling process, providing information on the degree of resin degradation and the transfer of soluble organic compounds to the aqueous phase. This analysis also enables the evaluation of the overall sustainability of the process, identifying the need for post-treatments or potential valorization routes for the effluents, aiming toward an integrated and sustainable composite recycling approach.

Table 4 summarizes the characterization of the liquid effluent from selected representative tests in terms of chemical oxygen demand (COD) and total organic carbon (TOC). The experiments carried out with water as solvent (LC2–LC5) showed an increase in both COD and TOC after treatment, consistent with the degradation of the epoxy matrix and the transfer of soluble organic fragments to the aqueous phase.

In the tests performed with pure isopropanol (LC13 and LC16), only a minor reduction in the organic load of the liquid effluent was observed, which is consistent with the gas-phase results, where gas generation was minimal under those conditions (see Figure 14).

By contrast, the run conducted with a 5% IPA solution at 550 °C (LC7) exhibited reductions of about 70% in both COD and TOC, suggesting a more extensive conversion of the organic matter than in water-based runs, likely due to the combined effect of the higher temperature and the reducing nature of the solvent, which promotes gasification/reforming reactions.

The catalytic experiments (LC21–LC23) also showed substantial reductions in organic load, particularly LC22 and LC23 (≥85%), consistent with the high gas yields observed in those runs (see Figure 14). In particular, LC22 exhibited a slightly higher reduction, attributable to the simultaneous generation of H_2_ and CO_2_, whereas LC23 displayed a greater selectivity toward H_2_, with a comparatively smaller carbon transfer to the gas phase. These results indicate that, under catalytic hydrothermal conditions, part of the dissolved organic matter is further converted into gaseous products, thereby decreasing the organic content of the liquid effluent.

The pH and conductivity values (Table 5) exhibited moderate variations between initial and final conditions. For the aqueous feeds, the initial pH (≈5.7) corresponds to deionized water equilibrated with atmospheric CO_2_ and the slight decreases after treatment can be attributed to the formation of weak organic acids during resin degradation. The largest change in conductivity was recorded in LC7. This is consistent with the aqueous, high-temperature setting (550 °C) promoting the formation and dissolution of ionic species in the liquid phase. By contrast, in the catalytic water–IPA runs (LC22–LC23), factors such as solvent composition, zinc/chloride speciation, and possible precipitation/complexation can limit the net increase in conductivity despite the high overall conversion.

In summary, the marked COD/TOC reductions achieved in the catalytic and high-temperature runs highlight the potential for integrated valorization of both gaseous and liquid streams in future designs of the hydrothermal carbon-fiber recycling process.

## 4. Conclusions and Perspectives

This study demonstrates the feasibility of hydrothermal solvolysis as an efficient and selective route for the recycling and valorization of carbon-fiber-reinforced polymers (CFRPs). Under the conditions explored, resin degradation rates up to ≈92% were achieved while preserving fiber morphology, sizing thickness, and mechanical performance (tensile strength and elastic modulus).

Temperature emerged as the primary driver of matrix decomposition, while reaction time had a limited effect once supercritical conditions were reached. The solvent-to-composite ratio and operating pressure correlated positively with degradation, although their effects are partially coupled, suggesting the need for more controlled experiments (e.g., semi-continuous testing).

The solvent played a decisive role. Water proved more effective than pure IPA under comparable conditions. Additionally, an unexpected and reproducible event (confirmed by several replicates) was observed when using a 40 wt.% IPA–water mixture, as follows: a sudden drop in temperature followed by a marked increase in pressure, which may have contributed to resin decomposition. Further work is required to clarify the underlying mechanism.

Based on the results of this work, water–IPA mixtures, used as a model of industrial wastewater streams, emerge as promising reaction media for hydrothermal recycling processes, enabling the simultaneous carbon fiber recovery and valorization of industrial wastewaters. Operationally, the use of IPA as a co-solvent helped to minimize fouling and facilitated cleaner runs. Introducing ZnCl_2_ as a homogeneous catalyst enabled high resin degradation while simultaneously generating H_2_-rich gas fractions (≈98 vol.% H_2_; ≈17 mol H_2_/kg) and achieving substantial COD/TOC reductions in the liquid. These findings reveal a dual opportunity for fiber recovery and byproduct valorization; however, ZnCl_2_ also induced severe corrosion phenomena on the 316 stainless-steel reactor, particularly under supercritical water conditions, highlighting the need to further investigate alternative, less aggressive catalytic systems for practical deployment.

The liquid stream can be valorized either as a feedstock to recover resin-derived chemicals, for the production of synthetic crude by hydrothermal liquefaction and/or direct liquid–liquid extraction, or as a reactive medium for supercritical water gasification (SCWG) to generate hydrogen, thereby enhancing overall energy integration. On the materials side, the recovered fibers can be used to manufacture recycled composites, parts, and prototypes.

Future work should prioritize the following: the optimization of catalytic formulations to lower operating temperature and intensify H_2_ production; the evaluation of semi-continuous configurations and heat/mass-integration strategies for scale-up; and application testing of the recovered fibers alongside fractionation or SCWG of liquid effluents to close the loop.

## Figures and Tables

**Table 1:** Characteristics of the two composite types used in this work.

Composite Type	SpA	SpB
Curing temperature (°C)	180	120
Dimensions (mm)	25 × 11 × 2	25 × 11 × 2
Weight (g)	0.5–1.0	0.5–1.0
Fiber type	AS4	T800S
Fiber diameter (µm)	7.1	5
Resin content (wt.%)	34 ± 2%	34 ± 2%

**Table 2:** Experimental conditions, and results in terms of decomposition rate.

Exp.	T [°C]	P [bar(a)]	t_r_ [min]	Solvent [wt.%]	Catalyst	P_0_ [bar(a)]	Ratio [mL/g]	Composite Type	SC	*DR* [%]
LC1	300	141	120	Water	-	37	92	SpA	N **	39
LC2	400	231	5	Water	-	11	164	SpA	Y **	80
LC3	400	231	35	Water	-	8	171	SpA	Y	78
LC4	400	241	35	Water	-	1	182	SpA	Y	87
LC5	400	231	60	Water	-	11	174	SpA	Y	82
LC6	425	251	35	Water	-	11	176	SpA	Y	78
LC7	550	231	40	IPA * 5%	-	1	159	SpB	Y	79
LC8	300	151	120	IPA 10%	-	37	236	SpA	N	29
LC9	300	156	35	IPA 20%	-	37	245	SpA	N	10
LC10	300	196	120	IPA 40%	-	37	234	SpA	Y	45
LC11	300	196	120	IPA 40%	-	37	228	SpA	Y	47
LC12	300	31	120	IPA	-	6	224	SpA	N	15
LC13	300	61	120	IPA	-	21	224	SpA	Y	11
LC14	450	111	40	IPA	-	12	170	SpA	Y	64
LC15	450	251	40	IPA	-	61	192	SpA	Y	67
LC16	450	251	40	IPA	-	61	220	SpA	Y	83
LC17	450	251	40	IPA	-	61	221	SpB	Y	80
LC18	300	141	120	Acet. 10%	-	38	233	SpA	N	33
LC19	420	251	40	Acet. 80%	-	66	176	SpA	Y	57
LC20	200	17	120	IPA 5%	ZnCl_2_ 0.1 M	1	192	SpB	N	2
LC21	300	88	120	IPA 5%	ZnCl_2_ 0.1 M	1	192	SpB	N	45
LC22	400	193	120	IPA 5%	ZnCl_2_ 0.1 M	1	193	SpB	N	78
LC23	415	233	120	IPA 5%	ZnCl_2_ 0.1 M	1	192	SpB	Y	92

* IPA (isopropanol, C_3_H_8_O). ** Y (solvent at supercritical conditions). N (solvent below supercritical conditions).

**Table 3:** Carbon fibers’ measured diameters and estimated sizing thicknesses.

Test	Original SpA	LC4	Original SpB	LC23
Average measured diameter (μm)	7.58 ± 0.2	7.36 ± 0.13	5.89 ± 0.04	5.94 ± 0.05
Average sizing thickness (μm)	0.25 ± 0.1	0.13 ± 0.07	0.45 ± 0.02	0.47 ± 0.03

**Table 4:** COD and TOC of the liquid effluents before (COD_0_, TOC_0_) and after (COD_f_, TOC_f_) the hydrothermal treatments.

Exp.	COD_0_ (mg O_2_/L)	COD_f_ (mg O_2_/L)	COD Removal (%)	TOC_0_ (mg C/L)	TOC_f_ (mg C/L)	TOC Removal (%)
LC2	0	1716	-	0	465	-
LC4	0	23,709	-	0	730	-
LC5	0	1898	-	0	508	-
LC7	102,947	31,570	69	26,915	9005	67
LC13	1,793,514	1,755,993	2	210,274	205,726	2
LC16	1,792,651	1,729,640	4	210,349	202,800	4
LC21	115,557	87,286	24	30,845	21,880	29
LC22	112,093	9875	91	29,430	2167	93
LC23	85,651	13,253	85	21,005	2937	86

**Table 5:** pH and conductivity values of liquid effluents.

Exp.	pH_0_	pH_f_	Conductivity_0_ (μS/cm)	Conductivity_f_ (μS/cm)
LC2	5.7	5.8	9	142
LC4	5.7	6.1	8	89
LC5	5.7	6.0	15	177
LC7	5.4	4.3	2	1019
LC21	5.8	5.7	15	17
LC22	6.0	5.5	15	21
LC23	5.9	4.7	17	22

## Data Availability

The datasets used and/or analyzed during the current study are available from the corresponding author on reasonable request.
